# Prediction of stroke patients’ bedroom-stay duration: machine-learning approach using wearable sensor data

**DOI:** 10.3389/fbioe.2023.1285945

**Published:** 2024-01-03

**Authors:** Takayuki Ogasawara, Masahiko Mukaino, Kenichi Matsunaga, Yoshitaka Wada, Takuya Suzuki, Yasushi Aoshima, Shotaro Furuzawa, Yuji Kono, Eiichi Saitoh, Masumi Yamaguchi, Yohei Otaka, Shingo Tsukada

**Affiliations:** ^1^ NTT Basic Research Laboratories and Bio-Medical Informatics Research Center, NTT Corporation, Atsugi, Japan; ^2^ Department of Rehabilitation Medicine I, School of Medicine, Fujita Health University, Toyoake, Japan; ^3^ Department of Rehabilitation Medicine, Hokkaido University Hospital, Sapporo, Japan; ^4^ NTT Device Innovation Center, NTT Corporation, Atsugi, Japan; ^5^ Department of Rehabilitation Medicine, Fujita Health University Hospital, Toyoake, Japan

**Keywords:** bedroom-stay duration, location tracking, rehabilitation, stroke, machine learning, wearable sensors

## Abstract

**Background:** The importance of being physically active and avoiding staying in bed has been recognized in stroke rehabilitation. However, studies have pointed out that stroke patients admitted to rehabilitation units often spend most of their day immobile and inactive, with limited opportunities for activity outside their bedrooms. To address this issue, it is necessary to record the duration of stroke patients staying in their bedrooms, but it is impractical for medical providers to do this manually during their daily work of providing care. Although an automated approach using wearable devices and access points is more practical, implementing these access points into medical facilities is costly. However, when combined with machine learning, predicting the duration of stroke patients staying in their bedrooms is possible with reduced cost. We assessed using machine learning to estimate bedroom-stay duration using activity data recorded with wearable devices.

**Method:** We recruited 99 stroke hemiparesis inpatients and conducted 343 measurements. Data on electrocardiograms and chest acceleration were measured using a wearable device, and the location name of the access point that detected the signal of the device was recorded. We first investigated the correlation between bedroom-stay duration measured from the access point as the objective variable and activity data measured with a wearable device and demographic information as explanatory variables. To evaluate the duration predictability, we then compared machine-learning models commonly used in medical studies.

**Results:** We conducted 228 measurements that surpassed a 90% data-acquisition rate using Bluetooth Low Energy. Among the explanatory variables, the period spent reclining and sitting/standing were correlated with bedroom-stay duration (Spearman’s rank correlation coefficient (R) of 0.56 and −0.52, *p* < 0.001). Interestingly, the sum of the motor and cognitive categories of the functional independence measure, clinical indicators of the abilities of stroke patients, lacked correlation. The correlation between the actual bedroom-stay duration and predicted one using machine-learning models resulted in an R of 0.72 and *p* < 0.001, suggesting the possibility of predicting bedroom-stay duration from activity data and demographics.

**Conclusion:** Wearable devices, coupled with machine learning, can predict the duration of patients staying in their bedrooms. Once trained, the machine-learning model can predict without continuously tracking the actual location, enabling more cost-effective and privacy-centric future measurements.

## 1 Introduction

### 1.1 Background and research aim

Several studies associated with stroke rehabilitation have reported a positive correlation between the duration of daily rehabilitation therapy and functional improvements in daily activities (Langhorne et al., 1996; Kwakkel et al., 2004; Yagi et al., 2017). It has also been expected that providing additional opportunities for physical activity, such as self-training and avoiding prolonged bed rest, supports better outcomes for patients (Patel et al., 2012; Tijsen et al., 2019). However, studies have shown that stroke patients admitted to rehabilitation units often spend a majority of their day in or near their beds (West and Bernhardt, 2013; Chouliara et al., 2021). One study highlighted that during most of their time in the bedroom, patients are either inactive or engaged in passive activities such as talking, reading, or watching television (West and Bernhardt, 2013). Such healthcare design can potentially impact patient outcomes or contribute to disuse syndrome (Li et al., 2020). Given the anticipated rise in global stroke-related deaths, advancing stroke rehabilitation to support better outcomes is a pressing issue globally (Fan et al., 2023). Wearable devices offer promising benefits for monitoring the activity of stroke patients (Rosenberger et al., 2016). They are increasingly used in medical studies in real-world settings due to their cost-effectiveness and small size. However, the complexity of impairment and disability in stroke patients poses challenges, especially regarding the detection accuracy of activity (Storti et al., 2008; Carroll et al., 2012). An alternative approach involves quantitatively assessing patient inactivity by recording the duration of stroke patients staying in their bedroom (West and Bernhardt, 2012). However, researchers conducted these observations manually, which may be impractical for healthcare providers to adopt in their routine care.

Automatically measuring the duration of stroke patients staying in their bedrooms using wireless access points that receive signals from patient-worn beacons or wearable devices will be useful. However, implementing access points in indoor environments, such as hospitals, requires a significant budget to cover expansive areas. To balance the cost-effectiveness of wearable devices with the accurate location-identification capabilities of access points, we focused on using machine-learning models, which are promising for the medical applications for their prediction and classification tasks, especially when paired with Internet of Things (IoT) devices (Kumar et al., 2023; Lai et al., 2023). Once trained via supervised learning using data from wearable devices, these models can potentially predict the duration of stroke patients staying in their bedrooms without continuous reliance on the actual location identified with access points. Predicting bedroom-stay duration may also support the planning of future care programs to obtain better outcomes.

Thus, our objective was to evaluate the performance of predicting the duration of stroke patients staying in their bedrooms using wearable-derived data. We also investigated the correlation between these estimates, demographic information, and patient-activity features measured with wearable devices.

### 1.2 Literature review

While the Global Positioning System or cellular wireless networks in wearable devices or smartphones is commonly used to identify location, it is often ineffective in indoor environments where electromagnetic shields, such as walls or doors, obstruct satellite or wireless signals (Hao et al., 2020). Attention has thus turned to indoor-positioning technologies, such as pedestrian dead reckoning (PDR), Wi-Fi, Bluetooth Low Energy (BLE), and radio frequency identification (RFID) (Wu et al., 2019; Alhomayani and Mahoor, 2020). We focused on long-term patient monitoring to measure the duration of stroke patients staying in their bedrooms for more than 24 h. Given that gyroscopes, essential for reliable PDR methods for estimating indoor location using only sensors (Hou and Bergmann, 2020), consume more power than other inertial sensors such as accelerometers, PDR is challenging for long-term monitoring. Hence, we opted for indoor-location tracking using BLE. BLE offers a balance between Wi-Fi and RFID, which is promising for reliable indoor-location tracking due to its wider wireless coverage than RFID and relatively lower power consumption than Wi-Fi, which is practical for clinical applications (Givehchian et al., 2022; Shang and Wang, 2022). There have been several reports on location-identification systems using BLE in hospitals (Rozum et al., 2019; Shipkovenski et al., 2020; Wichmann, 2022; Hadian et al., 2023), and in particular, systems that record patient locations covering their entire life space in hospitals in real-world settings have been developed (Abubeker and Baskar, 2023; Samama and Patarot, 2023). However, neither the automated recording of the duration of inpatients staying in their bedrooms nor its prediction has been demonstrated. To address this issue, we installed a system that simultaneously records both the activity and location of patients in hospital buildings. This system comprises a wearable device that sends BLE signals and access points installed on the ceilings of patients’ life spaces such as bedrooms, training rooms, and common spaces, enabling the automated recording of duration. This paper is the first study to explore the potential for machine learning to predict the duration of stroke patients staying in their bedrooms using activity data measured with a wearable device. To clarify the novelty of the present study, Table 1 summarizes the approaches of location-identification studies conducted in hospitals.

**TABLE 1:** Approaches of patient location-identification studies conducted in hospitals.

Study	Recording method of patient locations	Recording place in hospital	Experimental situation	Measurement of room-stay duration	Prediction of room-stay duration
West and Bernhardt (2013)	Manual	**Multi-location**	**Real world**	**Yes**	No
Chouliara et al. (2021)	Manual	**Multi-location**	**Real world**	**Yes**	No
Rozum t al. (2019)	**Automatic**	Single-location	**Real world**	No	No
Shipkovenski et al. (2020)	**Automatic**	**Multi-location**	Laboratory-testing	No	No
Hadian et al. (2023)	**Automatic**	Single-location	Laboratory-testing	No	No
Abubeker and Baskar (2023)	**Automatic**	**Multi-location**	**Real world**	No	No
Samama and Patarot (2023)	**Automatic**	**Multi-location**	**Real-world**	No	No
**Present study**	**Automatic**	**Multi-location**	**Real-world**	**Yes**	**Yes**

### 1.3 Paper structure

This paper is structured as follows. Section 2 presents participant details and the system designed to measure their locations and activities using BLE access points and wearable devices. This section also describes the activity features measured with wearable devices, machine-learning models, and statistical tests we used. Section 3 presents the results of activity measurements and location identifications and the evaluation of the prediction performance of the duration of stroke patients’ stay in their bedrooms. Section 4 discusses this study’s potential benefits and its limitations. Finally, Section 5 concludes the study.

## 2 Materials and methods

In this section, we detail the participants recruited for this study, devices integrated into the experimental system, signal processing of sensor data to compute features related to patient activity, machine-learning models used to estimate the duration of stroke patients staying in their bedrooms using these features, and statistical tests we conducted to verify significance.

### 2.1 Participants

We enrolled 99 hemiparetic stroke inpatients from Fujita Health University (FHU) Hospital. Written informed consent was obtained from all participants. We measured data on electrocardiograms (ECGs) and chest acceleration using a wearable device. The locations or names of the rooms where BLE access points detected the device’s signals emitted from the trunk of the participant, were recorded as well. Each measurement was conducted for 2 days. Patients underwent multiple measurement sessions with their consent; in such instances, these 2-day measurements were repeated biweekly. A total of 343 measurements were conducted. We also documented participants’ demographic information such as age, height, weight, and sex, as well as the motor and cognitive categories of the functional independence measure (mFIM and cFIM), which are clinical scales to assess the degree of independence of daily activities and communication (Keith et al., 1987). The mFIM and cFIM were assessed by rehabilitation professionals. On the basis of the data-acquisition rate, explained in a later section, 284 measurements involving 85 patients were selected and analyzed.

### 2.2 Experimental setup

**FIGURE 1:**
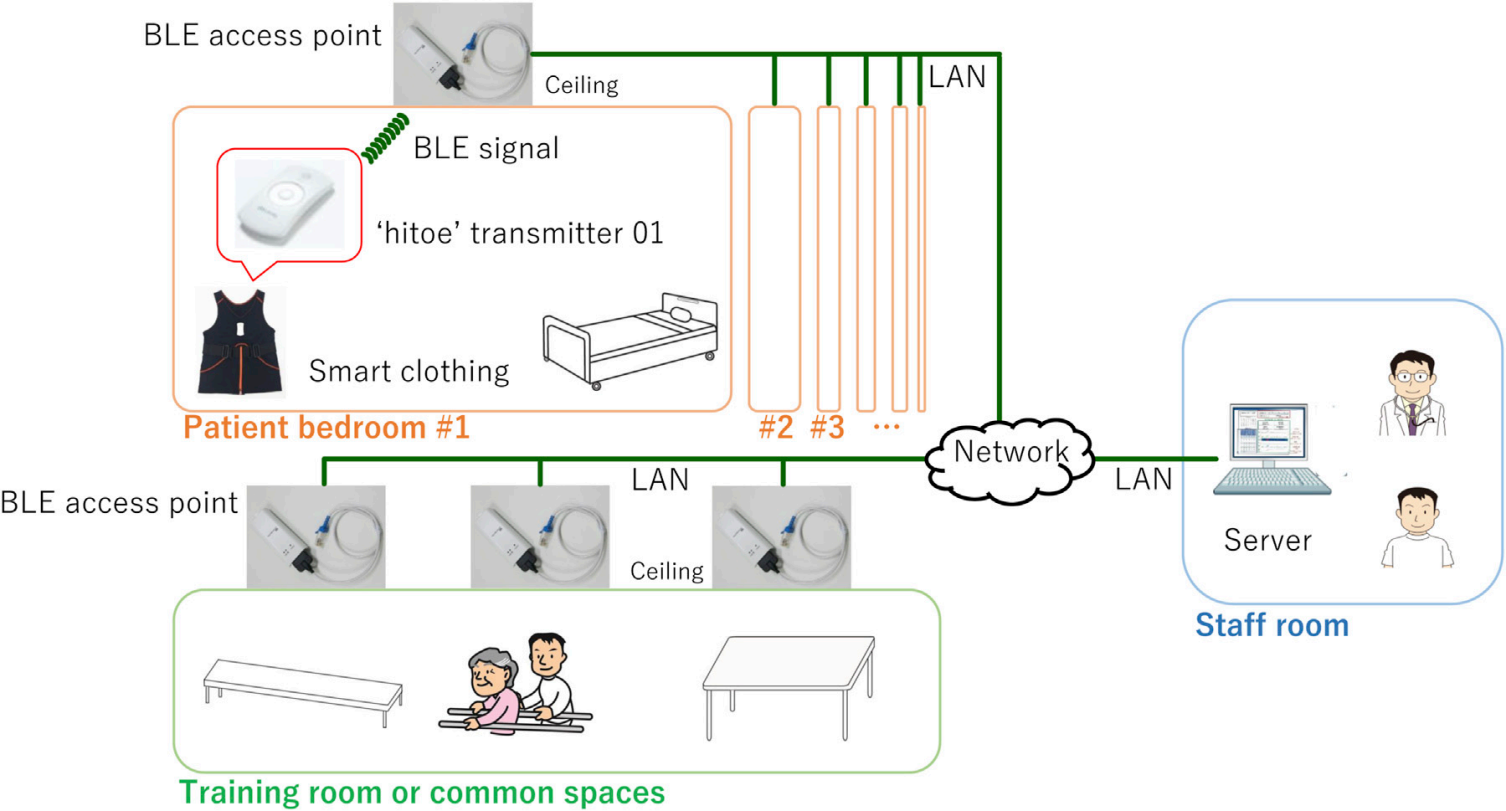
Configuration of experimental system. Wearable device, integrated into smart clothing, is worn by patient and equipped to send BLE signals. This device is used to continuously measure ECG and acceleration data of patient. BLE access points are installed throughout rehabilitation wards of FHU Hospital. Specifically, these access points are positioned on ceiling of each patient’s bedroom, training room, and common areas. These access points are designed to receive signals transmitted by wearable device worn by patient. Collected measurement data and location name are then transmitted and stored on server, which is located in staff room within hospital.

Figure 1 illustrates the configuration of the experimental system installed at FHU Hospital. The system comprises smart clothing with a transmitter that sends BLE signals and BLE access points. This clothing was worn by the patients, and BLE access points were positioned on the ceilings of each patient’s bedroom, the training room, and common areas for comprehensive coverage (Matsunaga et al., 2019). The “hitoe” transmitter 01 (NTT DOCOMO Inc., Tokyo) was used as the wearable device (Nakata et al., 2022). The smart clothing (Toray Industries, Inc., Tokyo) was designed to be skin-tight, ensuring the transmitter maintains close contact with the patient’s body; the clothing was changed daily. This device is equipped with an ECG and a three-axis acceleration sensor. The ECG and acceleration sensor have sampling rates of 1 kHz and 25 Hz, respectively (Tsukada et al., 2019; Ogasawara et al., 2021). To transfer the data measured with the wearable device to a server (PRIMERGY TX1320 M4, Fujitsu Ltd., Tokyo), we used a wireless gateway (OpenBlocks IoT BX0, Plat'Home Co., Ltd., Tokyo) as the BLE access point. These access points were networked with the server through the local area network (LAN) of FHU Hospital. We custom-developed the applications operating on the BLE access points and server using Python 3.9. We used PostgreSQL 13.6 to set up a database on the server, where the measurement data were stored.

**FIGURE 2:**
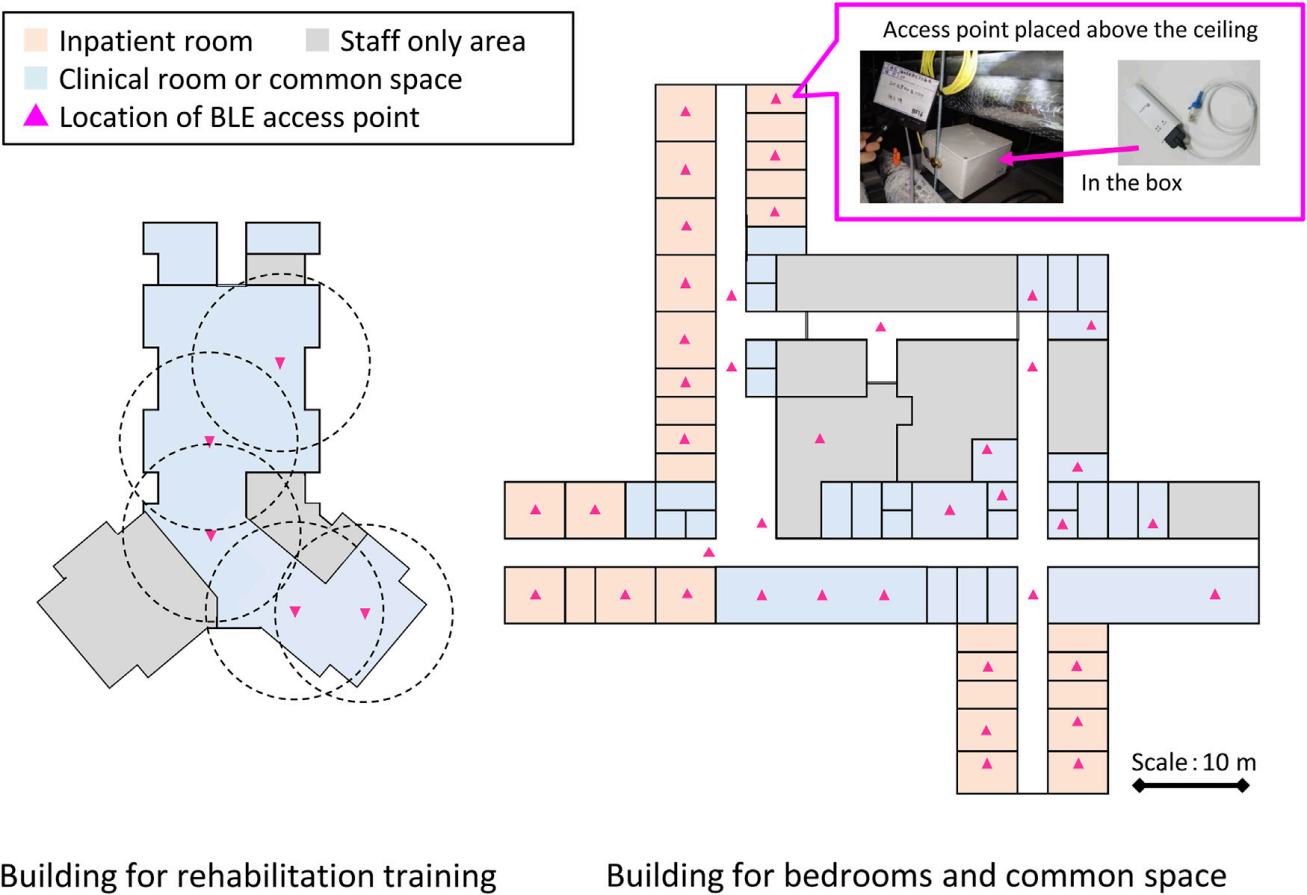
Location of BLE access points in rehabilitation wards. Approximately 50 BLE access points were distributed across two buildings to provide comprehensive coverage.

Figure 2 illustrates the placement of BLE access points in the rehabilitation wards. The FHU rehabilitation wards are located across two buildings: one building is dedicated to rehabilitation training activities, and the other is focused on the daily-life rehabilitation of inpatients. BLE signals, being generally weaker than Wi-Fi or cellular signals, present a unique challenge. Previous research indicates that BLE-signal strength attenuates significantly when a wearable device moves several meters from the access point or to an adjacent room separated by walls or doors (Faragher and Harle, 2015). This significant attenuation can lead to disconnection in wireless communication. To address this, we implemented the following two rules when deploying the access points within the buildings (Matsunaga et al., 2019). One is overlapping coverage. We aimed to keep the distance between neighboring BLE access points at about 5 m. This rule was intended to create overlapping zones of wireless coverage, ensuring that communication remains stable and continuous. Even in the spacious training areas, which lack substantial electromagnetic shielding, distances between access points were kept at less than 10 m. The other rule is dedicated bedroom coverage. We placed at least one access point in each patient’s bedroom. This rule was necessary due to the bedrooms in this hospital being electromagnetically shielded by metal-containing firewalls, which further dampen the already weak BLE signals. When an access point detects a BLE signal from a patient’s wearable device, it establishes a communication link with that device. All access points continuously monitor the received-signal-strength indicator of BLE advertise packets. If a patient moves and one access point loses the signal, another nearby access point is programmed to swiftly re-establish the connection with the wearable device to continue data acquisition. The data-acquisition rate between the BLE access points and wearable devices was found to be nearly identical to that with conventional monitoring approaches that use smartphones to connect with wearable devices (Matsunaga et al., 2019).

### 2.3 Signal processing to calculate features of stroke-patient activity

Table 2 lists the features of stroke-patient activity calculated from the measurement data acquired with a wearable device. The relationships between these features and the motor function of stroke patients have been explored (Arad et al., 2002; Kanai et al., 2018; Nathoo et al., 2018; Murayama et al., 2020; Mukaino et al., 2022). The experimental system described above automatically calculates these features using ECG and acceleration data. We briefly summarize this calculation process. The heart rate was calculated every minute using R-R (R-wave peak to R-wave peak) intervals detected in the ECG in the transmitter (Matsuura et al., 2022). Percent heart rate reserve (%HRR) is a well-known indicator that correlates with the amount of activity or exercise in stroke rehabilitation (Nathoo et al., 2018). The %HRR was calculated from heart rate and age in this study (Matsuura et al., 2019). Patient activity was classified into three categories: reclining, sitting/standing, and walking. This classification began with calculating the angle or declination of the acceleration sensor relative to the direction of gravitational acceleration in the sagittal plane of the patient’s trunk (Fortune et al., 2014). Using this calculated angle and a predetermined threshold, patients’ postures were initially categorized as either reclining or sitting/standing. To further delineate patient activity, the wearable device was designed to recognize periods when the patient was walking. This recognition was achieved by detecting walking steps, which were inferred from the norm of the acceleration using a rules-based algorithm (Ogasawara et al., 2016). Specifically, this allowed the system to differentiate between walking and non-walking states when the initial posture was identified as sitting/standing. This posture-classification method has been validated through studies involving both healthy individuals and clinical patients (Rauen et al., 2018; Ogasawara et al., 2023). In addition to posture classifications, moving standard averaging of trunk acceleration (MSDA) is used as an indicator of the physical-activity intensity of stroke patients (Mukaino et al., 2022).

**TABLE 2:** Features of stroke-patient activity calculated from measurement data of wearable devices.

Feature	Unit	Sensor
Heart rate	bpm	Electrocardiogram
% HRR	percent	Electrocardiogram
Period spent reclining	hour	Acceleration
Period spent sitting or standing	hour	Acceleration
Period spent walking	hour	Acceleration
MSDA	m/s^2^	Acceleration
Total number of walking steps	step	Acceleration

In recordings lasting more than 24 h, one of the challenges encountered is data loss. This loss can occur due to various factors including equipment failure, limitations in data-collection capabilities, and human error (Cismondi et al., 2013; Lai et al., 2020). To mitigate the impact of missing data, we implemented a data-imputation technique that is based on averaging (Ogasawara et al., 2021; Ogasawara et al., 2023). This technique generates a time series of features of stroke-patient activity over a 24-h period with reduced data loss by calculating the averages and ratios of these features at the same time of day during several measurement-session days.

### 2.4 Machine-learning models

We introduce a framework for estimating the bedroom-stay durations of inpatients by using the features of stroke-patient activity calculated from data measured with wearable devices in conjunction with machine-learning methodologies. The notation $Y_{i}^{\prime }\in R$ denotes the estimated room-stay duration, calculated with the features, while $Y_{i}\in R$ is the ground truth of the duration measured by access points. Here, *i* indexes the participants, ranging from 1 to *n*. For a successful estimation, the squared error $\sum_{i}{\left\|Y_{i}^{\prime }-Y_{i}\right\|}^{2}$ should be minimal, and a statistically significant correlation between $Y_{i}^{\prime }$ and $Y_{i}$ needs to be validated. The details of this validation process are described later in the paper. Given that $Y_{i}^{\prime }$ is calculated from the feature set **X**, it is represented as $Y_{i}^{\prime }=\Psi \left(X\right)$ , where **X** is a feature vector composed of *m* features obtained from data measured with wearable devices and represented as $X=\left(x_{1},x_{2},..,x_{m}\right)$ . When $t_{y}$ is the time recorded with access points and $Y_{i}$ is the total period of $t_{y}$ over 24 h, $Y_{i}=\sum t_{y}$ . Prediction of inpatient room-stay durations using wearables and machine-learning models becomes advantageous when $C\left(X\right)<\;C\left(t_{y}\right)$ holds, where $C\left(X\right)$ and $C\left(t_{y}\right)$ are the costs to acquire activity data of inpatients measured on wearables and location information logged on BLE access point, respectively, due to the cost of constructing access points or in scenarios where logging the location information is not permitted. In other words, hospitals that find it difficult to implement access points covering expansive areas may have the potential to quantitatively evaluate the duration.

**FIGURE 3:**
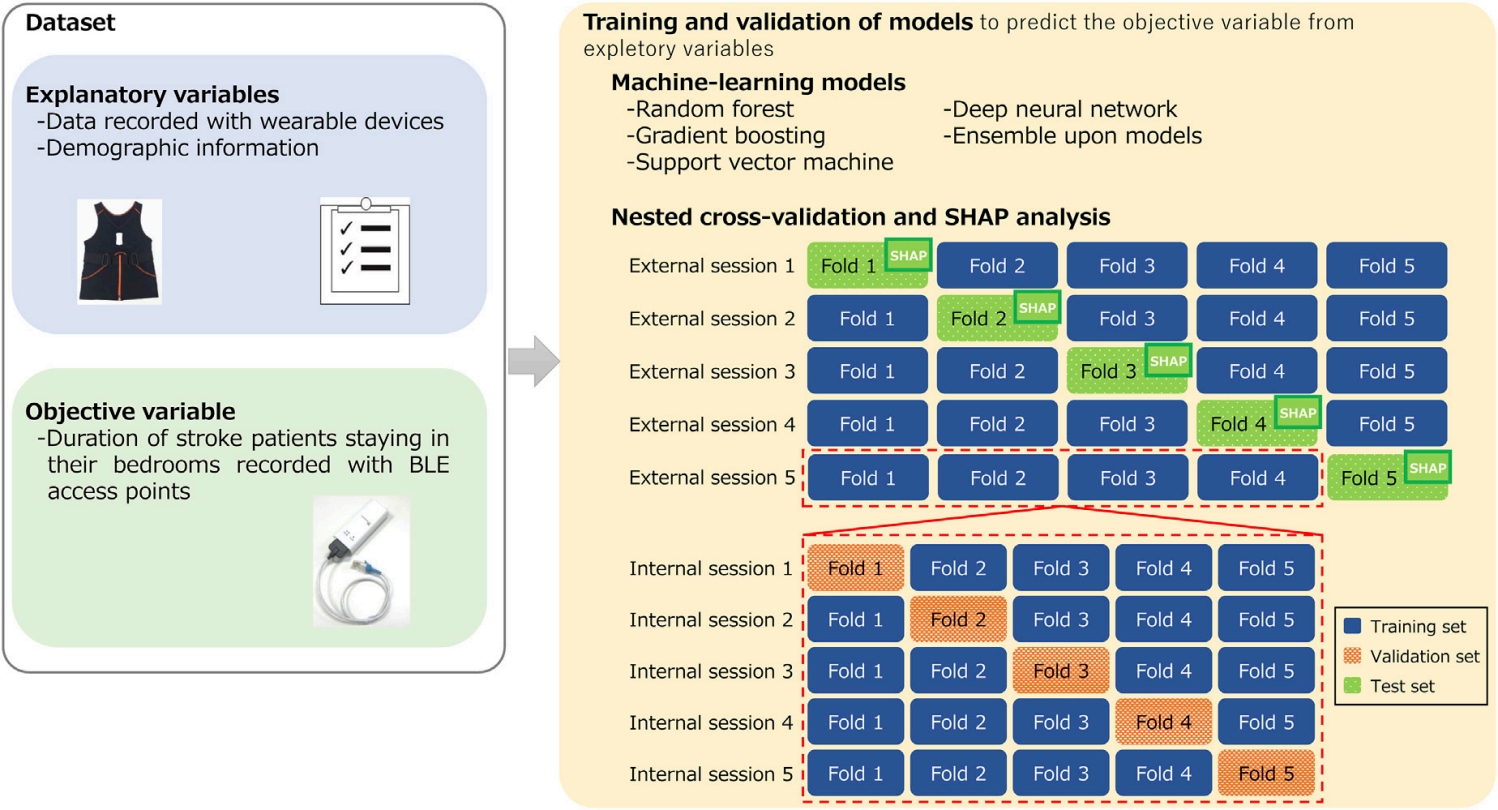
Training and validation processes undertaken with machine-learning models to estimate duration of stroke patients staying in their bedrooms.

Figure 3 illustrates the training and validation processes undertaken with machine-learning models. The objective variable to be estimated in this study was the duration of stroke patients staying in their bedrooms identified with BLE access points. The explanatory variables in the models include activity data measured with a wearable device, averaged over 24 h, and demographic information of the patients. We used a range of machine-learning models for comparison, including both established and newer ones. We used random forest (RF) (Dey et al., 2020), gradient boosting (GB) (Xu et al., 2022), and support vector machine (SVM) (Xiang et al., 2020), which are frequently used models in medical studies. These models were implemented using the scikit-learn library in Python 3. We also used a deep-neural-network (DNN) specifically designed for high-performance prediction of tabular data and implemented with the pytorch-tabular library (Joseph, 2021) as well as evaluated ensembles of these models.

To validate these machine-learning models, we used a nested cross-validation (CV), also known as double CV. This is a common approach to obtain robust prediction models and is effective at avoiding overfitting, which results in biased training outcomes (Cawley and Talbot, 2010; Vabalas et al., 2019). Nested CV consists of internal and external validation processes. The internal process aims to optimize the models’ hyperparameters. During this internal process, the set of hyperparameters that yield the lowest prediction errors are identified. With these optimal hyperparameters in place, the training and evaluation of the models are conducted in the external validation process using different test samples that were not involved in the internal process. CV provides a participant-independent estimate of performance for new or previously unseen participants because training and test dataset were different. In our study, samples were shuffled randomly at the starting of both internal and external CV. Both CVs were conducted with five folds. For hyperparameter optimization in the internal CV, we used AutoML from the Optuna library in Python 3 (Akiba et al., 2019).

To interpret the estimation mechanisms of the trained models, we used SHapley Additive exPlanations (SHAP) (Lundberg and Lee, 2017). SHAP is a unified framework for interpreting model predictions and enables us to investigate the contribution of each feature to the trained models. We conducted an overall feature summary analysis on the basis of SHAP.

### 2.5 Statistical analysis

To explore the relationships between the features calculated from the data recorded with the wearable device or demographics and the duration of stroke patients staying in their bedrooms, we conducted a statistical analysis. Initially, we conducted a Shapiro-Wilk test to assess the normality of the data distribution. If the normality of at least one of the paired values was not confirmed, a correlation analysis was carried out using Spearman’s rank correlation coefficient R (Schober and Vetter, 2020).

## 3 Results

This section first presents the results of a preliminary analysis then the demographic information of the participants involved in this study. The correlation relationships between the features of stroke-patient activity and demographics and the duration of the stroke patients staying in their bedrooms are also detailed. Finally, the evaluation of machine-learning models for estimating the duration of a patient staying in their bedroom is discussed.

### 3.1 Preliminary analysis

This section presents the assessment of BLE-access-point performance. Specifically, Section 3.1.1 assesses the performance of location identification using the BLE access points, and Section 3.1.2 assesses the data-acquisition rate of the BLE access points.

#### 3.1.1 Location identification using bluetooth low energy access points

To confirm the location-identification performance of the BLE access points, we assessed the duration for an access point to establish a connection with a wearable device when a user enters a room where the BLE access point is located as well as the duration for the connection to be terminated when a user exits the room (refer to Supplementary Figure S1). The results indicate that the average duration required to establish a connection with four participants was 8.82 s, and the average duration for disconnection was 1.03 s. The results suggest the sufficient specificity in the location-identification capabilities of the BLE access points because these durations are on the scale of seconds and fast enough for location data with a data-rate of 1 min. For further details, refer to Supplementary Method S1 and Results S1.

#### 3.1.2 Data-acquisition rate of bluetooth low energy access points

**FIGURE 4:**
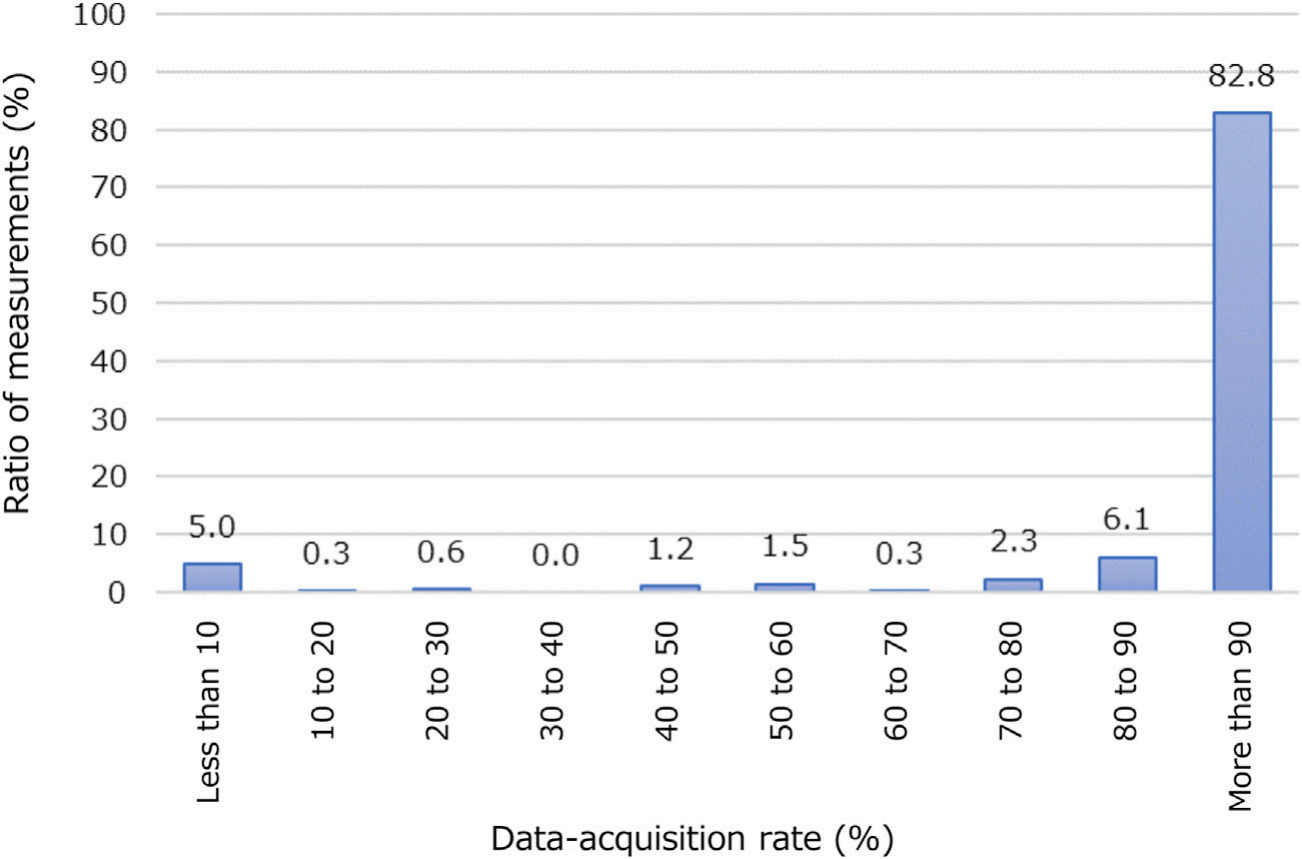
Acquisition rate of participants’ location data using BLE access points. Horizontal axis represents percentage of period when data of location information was successfully acquired at night (from 21:30 to 6:00). Vertical axis represents ratio of number of measurements in each class to total number of measurements (343 in this study).

Figure 4 illustrates the data-acquisition rate of participants’ location data using the BLE access points during nighttime hours (21:30 to 6:00). Nearly all patients are expected to remain in their bedrooms in this period. A 100% data-acquisition rate is achieved when there is no data loss between the wearable device and a BLE access point placed in rehabilitation wards. We set a 90% data-acquisition rate as the threshold for further analysis. Of the 343 measurements, 284 (or 82.8%) exceeded this threshold. The average data-acquisition rate among these 284 measurements was 98.0% ± 2.23%. We conducted correlation analysis and trained machine-learning models using these 284 measurements.

### 3.2 Patient demographics

Table 3 lists the demographics of the participants in this study. The sample size related to age, sex, height, and weight, corresponds to the number of patients (99 and 85). The sample size for other demographics corresponds to the number of measurements (343 and 284) because we repeated 2-day measurements biweekly for an identical patient, and these values often changed drastically.

**TABLE 3:** Patient demographics.

	343 measurements with 99 enrolled patients	284 analyzed measurements with 85 selected patients
Age (years)	70.9 ± 14.2	70.0 ± 14.5
Sex (male/female)	63/36	56/29
Height (cm)	161.5 ± 8.9	162.1 ± 8.5
Weight (kg)	56.0 ± 12.0	56.6 ± 12.4
Mobility (walk/wheelchair)	80/263	65/219
mFIM	48.2 ± 24.3	47.9 ± 24.5
cFIM	23.2 ± 9.4	23.1 ± 9.5
Duration of stroke patients staying in their bedrooms (hours)	15.2 ±4 .6	16.6 ± 3.2

mFIM, and cFIM, are clinical scales scored by rehabilitation professionals and used to assess degree of independence of daily activities and communication.

### 3.3 Correlation analysis

**FIGURE 5:**
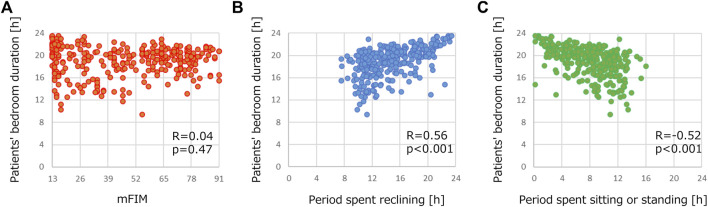
Examples of correlation plots of demographics in Table 3 and duration of stroke patients staying in their bedrooms. Correlation plots between patients’ bedroom duration and **(A)** mFIM, **(B)** period spent reclining and **(C)** period spent sitting or standing.

Table 4 shows the correlation relationships between the features of patient activity or demographic information and the duration of the patients staying in their bedrooms. We used R because a normal distribution was not confirmed with the duration of the patients staying in their bedrooms in the Shapiro-Wilk test. There were correlation relationships between the period spent reclining and the patients’ stay in their bedrooms (R = 0.56) and between the period spent sitting or standing and the time spent in their bedrooms (R = −0.52). Both relationships were statistically significant (*p* < 0.001). Interestingly, neither mFIM nor cFIM, clinical indicators of the motor or cognitive abilities of stroke patients, showed any correlation (R = −0.04 and 0.04). These results suggest the importance of monitoring patients’ activities using wearable devices. Figure 5 presents examples of correlation plots. Compared with the plot of mFIM, for which correlation was not confirmed, those of the period of reclining and sitting/standing displayed observable positive or negative slopes in distribution.

**TABLE 4:** Correlation relationships between features of stroke-patient activity or demographic information and duration of stroke patients staying in their bedrooms. The correlation relationships with statistical significance are highlighted in bold.

	Features	R	*p*-value
**Demographics of patient**	Elapsed week from admission	−0.18	0.002
	Age	0.14	0.02
	Sex	0.05	0.38
	Height	−0.04	0.49
	Weight	−0.05	0.39
	mFIM	−0.04	0.47
	cFIM	0.04	0.48
**Features of stroke-patient activity**	Heartrate	0.14	0.02
	%HRR	−0.07	0.24
	Period spent reclining	**0.56**	<0.001
	Period spent sitting or standing	**−0.52**	<0.001
	Period spent walking	−0.12	0.04
	MSDA	−0.15	0.01
	Walking steps	−0.12	0.04

### 3.4 Evaluation of machine-learning models for estimating stay duration in bedroom

**FIGURE 6:**
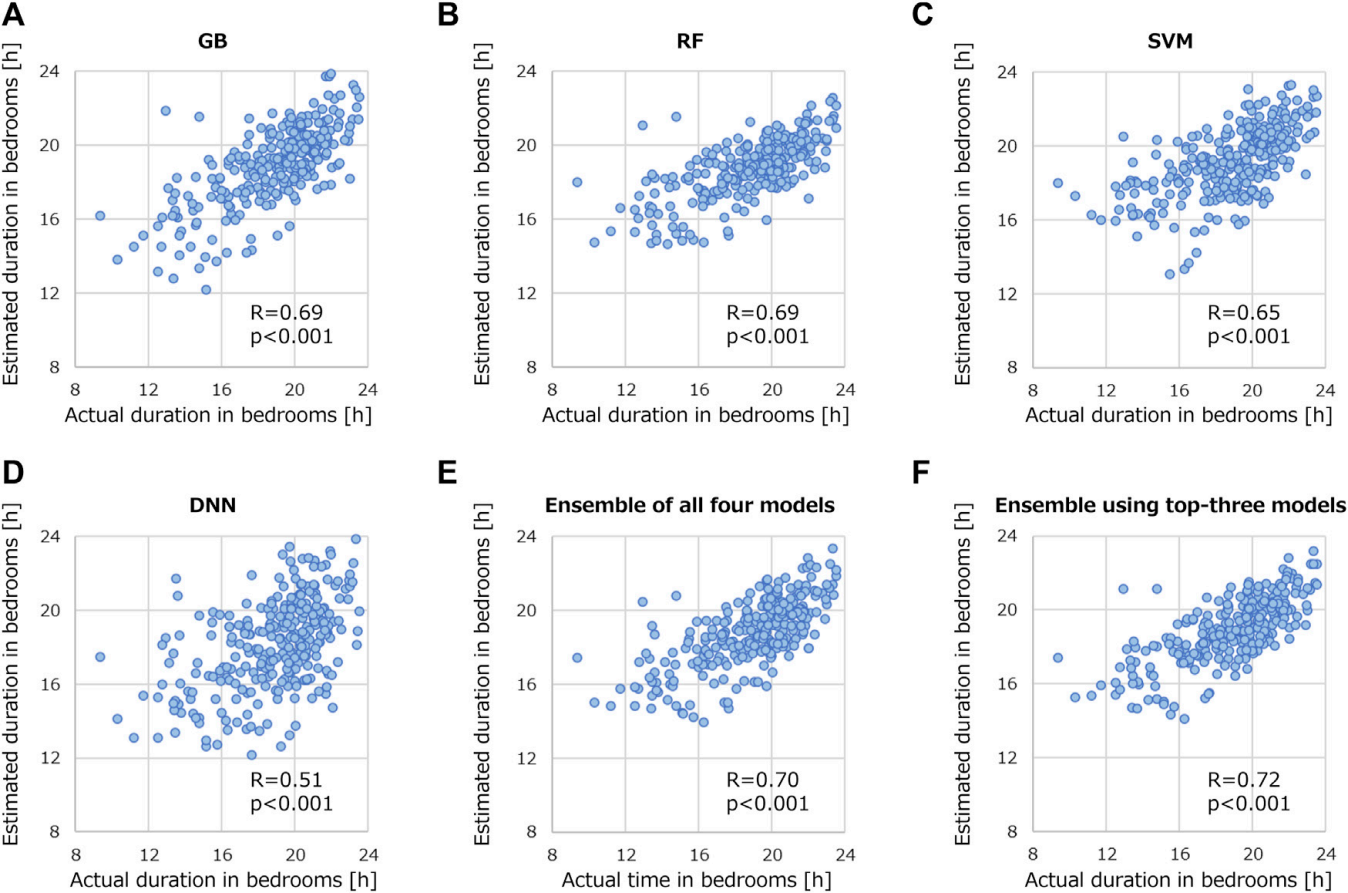
Correlation relationships between actual and estimated duration of stroke patients staying in their bedrooms. Validated models are **(A)** GB, **(B)** RF, **(C)** SVM, **(D)** DNN, **(E)** ensemble of all four models, and **(F)** ensemble using top-three models.

Figure 6 shows the correlation relationships between the actual and estimated durations of the patients staying in their bedrooms. The vertical axis represents the estimated duration identified with the BLE access points, and the horizontal axis represents the duration actual using machine-learning models. All results were statistically significant with *p*-values less than 0.001. Among the models in Figures 6A–D, R ranged from 0.51 to 0.69, with GB and RF proving the most successful in estimation (R = 0.69). Even the lowest R resulting from the DNN was close to the best R among features shown in the previous section (R = 0.51). When machine-learning models were ensembled, R further improved. Although R was 0.70 when all four models were ensembled (Figure 6E), excluding the DNN, R increased to 0.72 (Figure 6F). The results in Figure 6 support the validity of using machine learning to estimate the duration of stroke patients staying in their bedrooms.

**FIGURE 7:**
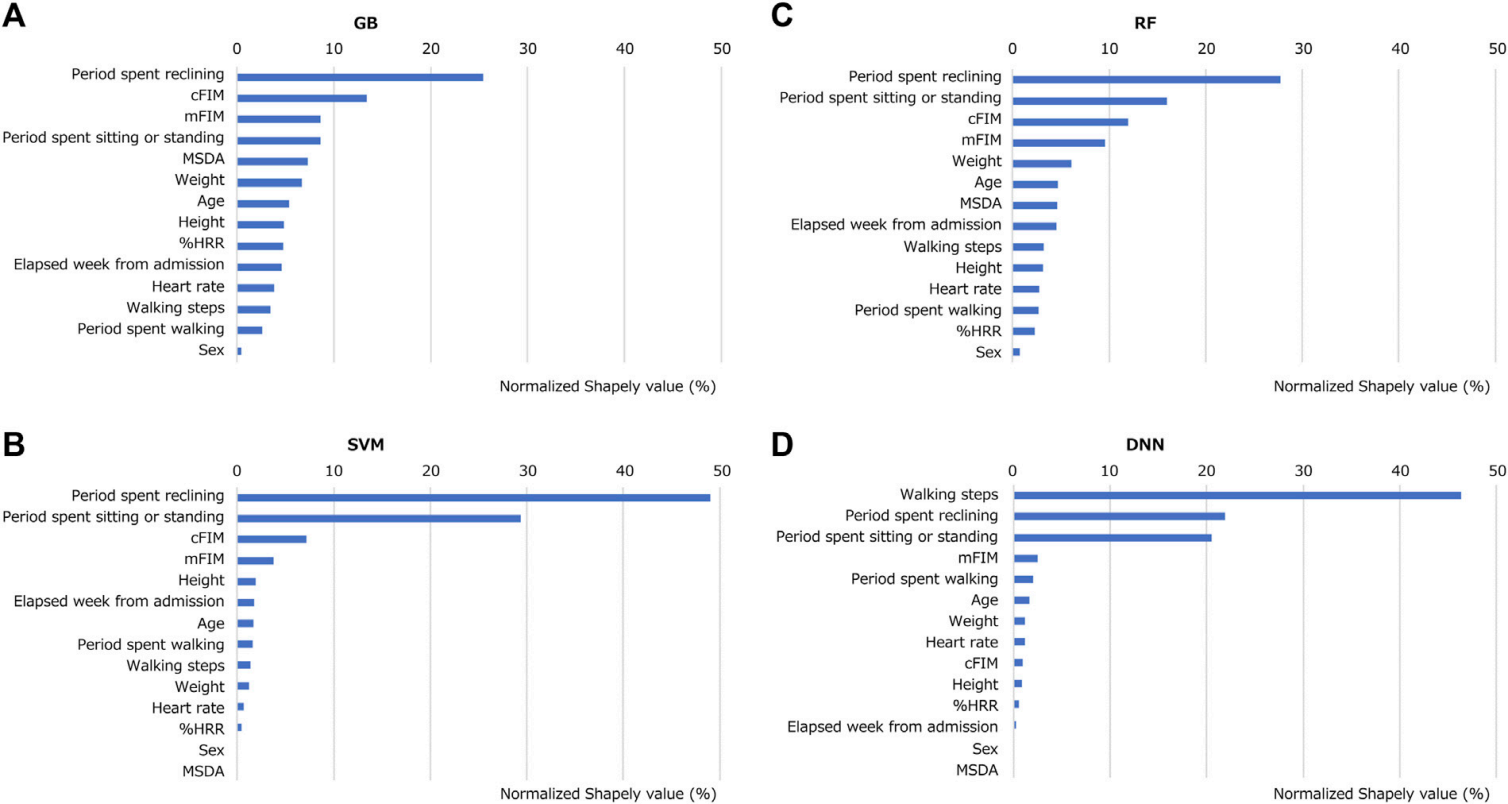
Contribution of each feature for trained models in predicting duration of stroke patients staying in their bedrooms. SHAP analysis was performed to the models of **(A)** GB, **(B)** SVM, **(C)** RF and **(D)** DNN.

Figure 7 presents the results of the SHAP analysis for the four machine-learning models. The horizontal axis of the plot displays the SHAP values, which quantify the contribution of each feature to the prediction performance of a model. These SHAP values are normalized and presented as a percentage of the total contribution of all features. In all the models except the DNN, the period spent reclining emerged as the feature with the most substantial contribution to the predictions regarding the duration of stroke patients staying in their bedrooms. Other notable contributors across the models were the periods spent sitting/standing, mFIM, and cFIM. Interestingly, in the DNN, the number of walking steps showed the highest contribution, differing from the other models.

**FIGURE 8:**
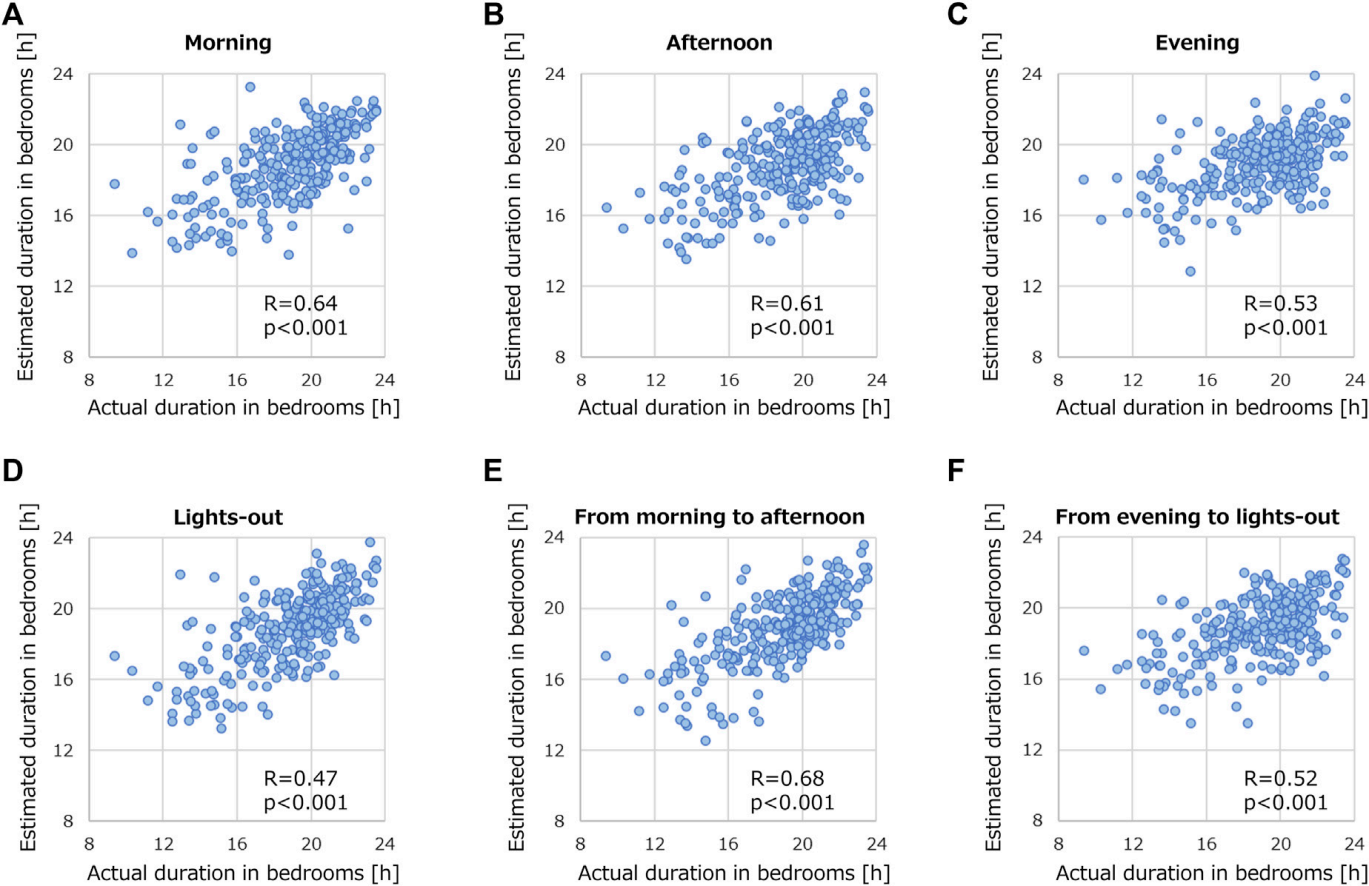
Correlation relationships between actual and estimated duration of stroke patients staying in their bedrooms by using recorded data. The data was segmented into various time periods: **(A)** morning (6:00 to 13:00), **(B)** afternoon (13:00 to 18:00), **(C)** evening (18:00 to 21:30), **(D)** lights-out (21:30 to 6:00), **(E)** morning to afternoon (6:00 to 18:00), and **(F)** evening to lights-out (18:00 to 6:00). GB was employed for this analysis.

We conducted another investigation into how the recording time-window impacts the prediction performance of the models. Figure 8 presents the prediction performances of GB using data measured during different segments of the day: morning (6:00 to 13:00), afternoon (13:00 to 18:00), evening (18:00 to 21:30), and lights-out period (21:30 to 6:00). Using only data recorded in the morning or afternoon (Figures 8A,B) yielded notable correlations: R of 0.64 and 0.61, respectively. However, these correlations fell short compared with that using record during 24 h R = 0.69 (Figure 6A). Predictions based on data recorded in the evening or lights-out period were even less successful, as shown in Figures 8C,D. When the time window was expanded to encompass both morning and afternoon (Figure 8E), R peaked at 0.68. In contrast, R remained low on the basis of data recorded both in the evening and lights-out period (Figure 8F). These results suggest that data collection only in daytime is helpful, and a broader recording window results in developing better models to predict the duration of stroke patients staying in their bedrooms.

## 4 Discussion

Our study advanced prior research in two ways. We first demonstrated the automatic recording of the duration of stroke patients staying in their bedrooms by implementing a location-identification system with BLE signals. We then suggested the possibility of predicting the duration using wearable-derived data. We now discuss the potential advantages and limitations of predicting the duration of stroke patients staying in their bedrooms using a wearable device and machine learning, which will lead to future work.

### 4.1 Potential advantages

#### 4.1.1 Feature analysis

We investigated features that could be related to the duration of a patient staying in their bedroom. As indicated in Table 4, significant correlations were suggested between the period spent reclining and those spent sitting/standing. In contrast, demographic information showed no significant relationship. Thus, the results suggest the importance of activity measurement with a wearable device. Those features indicating posture were important valuables for the machine-learning models, as shown in Figure 7, and were calculated from acceleration data. An acceleration-sensor module is generally power-efficient and cost-effective, making it suitable for long-term activity monitoring.

Among the clinical scales for rehabilitation, mFIM and cFIM demonstrated a lack of strong correlation with the bedroom stay duration. They are used to evaluate independence in terms of need for assistance with daily activities and communication scored by rehabilitation professionals. Previous studies have suggested that many stroke patients often spend a majority of their day in or near their beds regardless of their FIM values (West and Bernhardt, 2013; Chouliara et al., 2021). The results from our study support the tendency. However, mFIM and cFIM exhibited significant contributions in the SHAP analysis in tree-structured models, such as GB and RF. This finding suggests that these machine-learning models might assign importance to mFIM and cFIM not linearly related. In the tree-structured models, for example, these non-correlating indicators might prove valuable in deeper branches for estimation.

#### 4.1.2 Machine learning

Some machine-learning models yielded higher R of 0.69, as shown in Figures 6A,B, compared with the best R of 0.56 derived from the patient-activity features presented in Table 4. This suggests the potential usefulness of machine-learning models to predict the duration of patients staying in their bedrooms. It also suggests the possibility of conducting more cost-effective and privacy-conscious measurements in the future, as once the model has been trained with supervised learning, it can make predictions using only the explanatory variables without further recording and storing the patients’ actual location history.

The technique used to combine prediction models demonstrated enhanced prediction performance, as shown in Figure 6. This is consistent with theoretical studies that indicate an appropriately constructed ensemble model typically offers a reduced squared error compared with individual predictive models (Krogh and Vedelsby, 1994; Ganaie et al., 2022). We experimentally applied the theory in activity monitoring. To construct an appropriate ensemble model, we eliminated the poor-prediction model, as shown in Figure 6F. The ensemble technique we used was quite basic, averaging the predicted values of the models. This suggests the potential for enhanced performance by adding more sophisticated techniques, such as attention models (Singh et al., 2023), in future research.

#### 4.1.3 Measurement time of wearable devices

By using only the activity data recorded from morning to afternoon, we achieved a prediction performance (R = 0.68 in Figure 8E) that was closely comparable to using data from the entire day (R = 0.69 in Figure 6A). This suggests that the time during which activity is recorded plays a crucial role in predicting the duration of a patient staying in their bedroom. The possible reason could be that many patients tend to stay in their bedrooms from evening to the next morning, regardless of their physical condition. As wearable devices often require close body contact, which some patients find it uncomfortable, using such devices in the daytime might be a practical solution.

### 4.2 Limitations and future work

#### 4.2.1 Data loss of location tracking

While using BLE access points for location tracking within the rehabilitation wards, we found that only 284 out of the 43 measurements (82.8%) achieved a data-acquisition rate exceeding 90%. This indicates that data loss remains a challenge that needs addressing, which is similar to other medical-sensor-network systems (Selvarajan et al., 2023). This data loss could be due to equipment malfunctions, such as battery depletion, interference from electromagnetic shields in the environment, and human errors. Specifically, BLE communication is prone to disruptions because of issues such as multipath fading and fluctuating conditions (Iannizzotto et al., 2023). Machine-learning techniques to impute missing health records have been proposed and could prove beneficial for studies that rely on location tracking (Getz et al., 2023).

#### 4.2.2 Prediction performance of machine-learning models

The predictive capabilities of the machine-learning models we used, especially the DNN, still need to be addressed. The performance of the DNN (R = 0.51) was lower than those of the more common, well-established models of GB, RF and SVM. This is consistent with previous studies that the best performance with tabular data is often achieved with “shallow” models (Joseph, 2021). One potential explanation for this discrepancy might be the limited size of our dataset. Deep learning typically requires large datasets, often spanning hundreds or even thousands of examples. Fields, such as microarray studies or gene analyses, often have access to such voluminous data, but for many fields, accumulating sizable datasets remains a challenge (Park and Tamura, 2019; Lin and Tsai, 2020). Augmenting sensor data is also challenging, unlike using image data, where augmentation is a commonly used strategy to expand datasets. Thus, using an ensemble of models, as was done in this study, may be crucial with smaller datasets. Another alternative could be to transfer learning and self-supervised learning known to be tunable even with small datasets (Ebbehoj et al., 2022; Rani et al., 2023).

#### 4.2.3 Feature exploring and bias of dataset

While we evaluated 14 features for our machine-learning models, there remain additional features yet to be explored. We could not include features related to heart rate variability (HRV) due to our experimental system’s data-transfer limitations. Given the advancements in recent studies on the capability to monitor HRV features, we anticipate their inclusion in future research (Ashokkumar et al., 2023). Assessing a patient’s in-bed status and their restlessness during rest are also important from a clinical standpoint. A broader range of sensors is essential to cover such features. Moving forward, it will be important to increase the number of sensor types and leverage sensor-fusion methodologies (Khadidos et al., 2023).

Data bias is also a limitation. All data for this study were collected in a single facility, which might not capture the variability of data from diverse demographics. To develop a more applicable model, data from multiple facilities would be crucial. However, the implementation of BLE access points across numerous locations may not be financially feasible for many hospitals due to hardware and installation costs. Given that the need for actual location data is temporal during the model-training phase, dozens of facilities would be sufficient. Follow-up studies at multiple facilities are needed to verify these possibilities.

## 5 Conclusion

We investigated the prediction of the duration of patients staying in their bedrooms using machine learning and wearable devices. We analyzed 284 measurements among 343, which exceeded the 90% data-acquisition rate at BLE access points. Correlation analysis suggested that a wearable device could provide indicators related to the duration of patients staying in their bedrooms, and those were the periods spent reclining and sitting/standing with an R of 0.56 and −0.52. Interestingly, mFIM or cFIM did not show any correlation. The machine-learning models RF, GB, SVM, DNN, and an ensemble of them were evaluated. The highest R among these models was 0.72, suggesting the possibility of predicting the duration of patients staying in their bedrooms from activity data and demographic information. In SHAP analysis, the machine-learning models showed high contribution to features with/without correlations, which were the period spent reclining and sitting/standing, mFIM, cFIM, and walking steps. Using only the activity data recorded from morning to afternoon, the prediction performance was R = 0.68, suggesting measurement during only the daytime may be useful. These results suggest the possibility of conducting more privacy-conscious measurements, as once the model has been trained with supervised learning, it can make predictions using only the explanatory variables without further recording and storing the patients’ actual location history.

## Data Availability

The raw data supporting the conclusion of this article will be made available by the authors, without undue reservation.
