# Supplementary material for: Prediction of stroke patients’ bedroom-stay duration: machine-learning approach using wearable sensor data
Source: Front Bioeng Biotechnol. 2024 Jan 3;11:1285945. doi: 10.3389/fbioe.2023.1285945 (PMC10791943; doi:10.3389/fbioe.2023.1285945)
Supplement: Supplementary file 1 [file DataSheet1.PDF]

Supplemental method 1 and results 1

We examined the time it takes for an access point to establish a connection with a wearable device when a user enters a room as well as the time it takes for the connection to be terminated when a user exits the room.

1 Supplemental Method 1

Four healthy participants participated in this study. The participants agreed to wear the wearable devices and enter or exit a room where a BLE access point was located. Each participant did this test ten times, and the durations for both connection and disconnection were measured. The experiment was conducted in a fireproof facility involving metal materials (which behave as electro-magnetic shields) as well as patient bedrooms at FHU Hospital.

2 Supplemental Results 1 and Discussion

Supplemental Figure 1 reveals that the average duration for establishing a connection with the four participants was 8.82 ± 0.57 seconds, while the average duration for disconnection was 1.03 ± 0.22 seconds. These durations are on the scale of seconds and sufficiently rapid for location data with a data-rate of 1 minute. This suggests the specificity of the location-identification capability of the BLE access point.

The duration until disconnection was found to be shorter than that until connection. This is likely because the disconnection event is more readily noticeable with missing packet—occurring when the BLE signal becomes unreachable. In contrast, the connection process requires two steps: initially finding an advertisement packet and subsequently establishing a communication link.

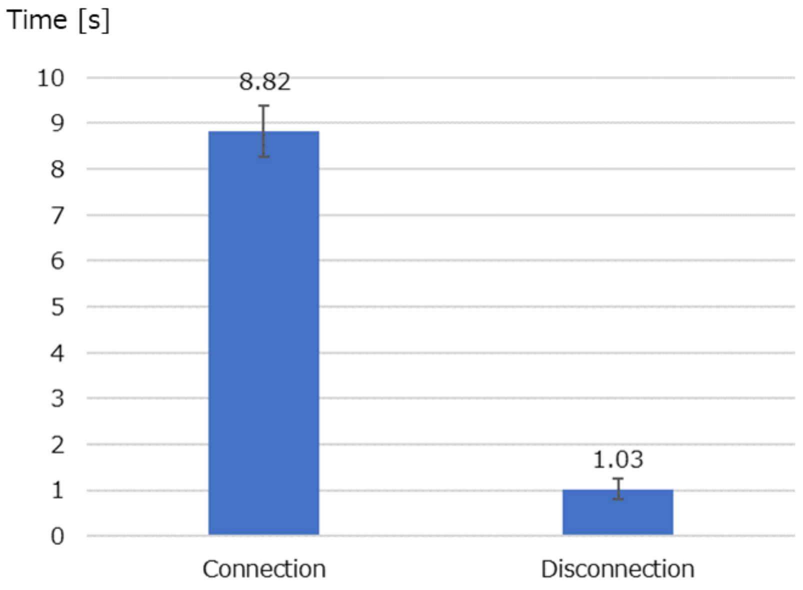

Supplemental Figure 1. Averages duration until connection and disconnection (n=4).
